# Knot selection in sparse Gaussian processes with a variational objective function

**DOI:** 10.1002/sam.11459

**Published:** 2020-04-20

**Authors:** Nathaniel Garton, Jarad Niemi, Alicia Carriquiry

**Affiliations:** ^1^ Department of Statistics Iowa State University Ames Iowa USA

**Keywords:** knot selection, machine learning, nonparametric regression, sparse Gaussian processes, variational inference

## Abstract

Sparse, knot‐based Gaussian processes have enjoyed considerable success as scalable approximations of full Gaussian processes. Certain sparse models can be derived through specific variational approximations to the true posterior, and knots can be selected to minimize the Kullback‐Leibler divergence between the approximate and true posterior. While this has been a successful approach, simultaneous optimization of knots can be slow due to the number of parameters being optimized. Furthermore, there have been few proposed methods for selecting the number of knots, and no experimental results exist in the literature. We propose using a one‐at‐a‐time knot selection algorithm based on Bayesian optimization to select the number and locations of knots. We showcase the competitive performance of this method relative to optimization of knots simultaneously on three benchmark datasets, but at a fraction of the computational cost.

## 1 INTRODUCTION

Gaussian processes (GPs) are a class of Bayesian nonparametric models with a plethora of uses, such as nonparametric regression and classification, spatial and time series modeling, density estimation, and numerical optimization and integration. Their use, however, is restricted to small datasets due to the need to store and invert an *N* × *N* covariance matrix, where *N* is the number of observed data points. This leads to storage scaling $O\left(N^{2}\right)$ and computation time scaling $O\left(N^{3}\right)$.

To address these computational challenges, there has been a large amount of literature on certain approximations to GPs, commonly called *sparse* GPs, which achieve linear storage and time complexity in *N* [5, 7, 19, 20, 21, 25]. Many of these methods rely on a subset of input locations, which we refer to as knots, to induce marginal covariances between function values. Models are defined so that the inverse of the approximating *N* × *N* covariance matrix, also called the precision matrix, is sparse. That is, most of the elements of the precision matrix are zero, hence the justification for the name “sparse” GPs.

Despite the success of these methods, one significant challenge in practice is selecting the number and locations of knots. One currently very popular practice is to optimize a predefined number of knots simultaneously alongside covariance parameters with respect to some objective function using continuous optimization. The two most common objective functions are the marginal likelihood (or an approximation of it) [4, 12, 15, 21] and the evidence lower bound in case a variational inference approach is taken [4, 11, 22]. While this is often successful in practice, it requires the user to choose the number of knots, *K*, up front. One can opt to make *K* as large as is computationally feasible, but this may not always be necessary to achieve accurate predictions; we will demonstrate this on some real data experiments. Furthermore, as we will show, the computational burden associated with the continuous optimization may grow substantially due to a large number of additional parameters associated with the knots.

Reference [8] proposed an efficient one‐at‐a‐time (OAT) knot selection algorithm based on Bayesian optimization to select the number and locations of knots in sparse GPs when the objective function is the marginal likelihood. One aim of their algorithm was to mitigate optimization issues often encountered when using the marginal likelihood as the objective function. However, they also found that, even when the aforementioned optimization issues were not substantial, the OAT algorithm was able to effectively select knots so that the resulting models were competitively accurate compared to performing simultaneous optimization. Furthermore, the OAT algorithm tended to be several times faster than simultaneous optimization.

In this paper, we extend the use of the novel OAT knot selection algorithm in ref. [8] to the context of nonparametric regression and variational inference. We provide experimental results on three real datasets showing the competitive accuracy of models selected using the OAT algorithm compared to those chosen via simultaneous optimization, but often at a lower computational cost. We also compare the performance of the OAT algorithm when used with the evidence lower bound versus with the marginal likelihood as the objective function.

The remainder of this paper is as follows. In Section 2, we briefly introduce GP regression. Section 3 introduces the class of knot‐based sparse GPs that we consider. Section 4 describes variational inference generally and in the context of the relevant sparse GP models. We also discuss some details regarding the evidence lower bound as the knot selection objective function, and we provide an illustrative, one‐dimensional regression example. In Section 5, we show experimental results on three benchmark datasets, and in Section 6, we conclude with a discussion.

## 2 GP REGRESSION

We assume that we have *N* observations, ${\left\{\left(y_{i},x_{i}^{⊤}\right)\right\}}_{i=1}^{N}$, from a dataset where each *y*_*i*_ ∈ ℝ is the target of interest, and the values *x*_*i*_ are vectors of input variables where $x_{i}\in X$ and X is a compact subset of ℝ^*d*^. We suppose that, over X_,_ there is an unobservable, real‐valued function f:X→ℝ taking values *f*(*x*_*i*_). We further suppose that the values of this function give the mean of the (conditional) distribution of the target random variable *Y*_*i*_ and that the *Y*_*i*_ random variables are conditionally independent given the *f*(*x*_*i*_). That is, we assume

$$Y_{i}|f\left(x_{i}\right)\overset{\mathrm{ind}}{\text{∼}}N\left(f\left(x_{i}\right),\tau^{2}\right),$$

where *τ*^2^ is variance due to random noise. Note that *τ*^2^ is also sometimes called a *nugget*.

We can use a GP as a prior distribution on the latent function. We denote this as $f\left(x\right)∼\mathrm{GP}\left(m\left(x\right),k_{\theta }\left(x,x^{\prime }\right)\right)$, where *m*(*x*) is the mean function, and $k_{\theta }\left(x,x^{\prime }\right)$ is the covariance function. We assume the covariance function is parameterized by *θ*. We will use $x={\left\{x_{i}\right\}}_{i=1}^{N}$ to denote the set of observed input locations, and we will use $\tilde{x}={\left\{{\tilde{x}}_{i}\right\}}_{i=1}^{J}$ to denote unobserved input locations at which we wish to predict the corresponding target values. The difference between x and $\tilde{x}$ is that $f_{\tilde{x}}$ depends on *Y* only through $f_{x}$. A GP, by definition, is a collection of random variables such that any finite subcollection $f_{x^{\prime }}={\left(f\left(x_{1}^{\prime }\right),\cdots ,f\left(x_{M}^{\prime }\right)\right)}^{⊤}∼N_{M}\left(m_{x^{\prime }},\sum_{x^{\prime }x^{\prime }}\right)$, where $m_{x^{\prime }}={\left(m\left(x_{1}^{\prime }\right),\cdots ,m\left(x_{M}^{\prime }\right)\right)}^{⊤}$ and the *ij*th element of $\sum_{x^{\prime }x^{\prime }}\left(i,j\right)=k_{\theta }\left(x_{i}^{\prime },x_{j}^{\prime }\right)$. In general, we will use notation $\sum_{{\mathrm{xx}}^{\prime }}$ to denote the matrix of covariances between elements of $f_{x}$ and $f_{x^{\prime }}$, where *ij*th element of $\sum_{{\mathrm{xx}}^{\prime }}\left(i,j\right)=k_{\theta }\left(x_{i},x_{j}^{\prime }\right)$.

Our assumed data model implies the following joint distribution for ${\left(Y^{⊤},f_{x}^{⊤}\right)}^{⊤}$,

$$\left[\begin{array}{c} Y\\ f_{x} \end{array}\right]∼N\left(\left[\begin{array}{c} m_{x}\\ m_{x} \end{array}\right],\left[\begin{array}{cc} \sum_{\mathrm{xx}}+\tau^{2}I & \sum_{\mathrm{xx}}\\ \sum_{\mathrm{xx}} & \sum_{\mathrm{xx}} \end{array}\right]\right).$$

Similarly, we can write down the distribution for ${\left(Y^{⊤},f_{\tilde{x}}^{⊤}\right)}^{⊤}$, which is

$$\left[\begin{array}{c} Y\\ f_{\tilde{x}} \end{array}\right]∼N\left(\left[\begin{array}{c} m_{x}\\ m_{\tilde{x}} \end{array}\right],\left[\begin{array}{cc} \sum_{\mathrm{xx}}+\tau^{2}I & \sum_{x\tilde{x}}\\ \sum_{\tilde{x}x} & \sum_{\tilde{x}\tilde{x}} \end{array}\right]\right).$$

GP prediction works by formulating the conditional distribution of $f_{\tilde{x}}|Y$, which, using standard rules regarding multivariate Gaussian distributions, is the following

$$\begin{array}{cc} & f_{\tilde{x}}|Y∼N\left(m_{\tilde{x}}+\sum_{\tilde{x}x}{\left(\sum_{\mathrm{xx}}+\tau^{2}I\right)}^{-1}\left(y-m_{x}\right)\right.\\ & \;\left.\;\sum_{\tilde{x}\tilde{x}}-\sum_{\tilde{x}x}{\left(\sum_{\mathrm{xx}}+\tau^{2}I\right)}^{-1}\sum_{x\tilde{x}}\right). \end{array}$$

## 3 SPARSE, KNOT‐BASED GPs

We discussed that GPs can be used as a prior distribution over functions. Importantly, however, GPs only directly impact inferences through a finite dimensional marginal distribution on relevant function values. Sparse GPs are also used as prior distributions over the same relevant finite set of function values, but they have more appealing computational properties than full GPs [16]. Some, but not all, sparse GPs correspond to true functional priors [16]. Thus, sparse GPs are prior distributions that approximate the ideal, full GP prior. We explain this more precisely in the following paragraphs. It is worth noting that, ordinarily, the *posterior* distribution of the latent function is of more interest than the prior. The variational inference method of [22] that we discuss in Section 4.1 directly specifies an approximation to the posterior of a full GP, which corresponds to the approximate posterior resulting from one of the sparse priors discussed in this section. We will explain this in detail in Section 4.1.

The sparse GPs that we consider are all based on the assumption that, conditional on a small subset of function values, the remaining function values in the *training set* are independent. The input locations corresponding to this small set of function values have variously been referred to as knots [1, 7], pseudoinputs [21], or inducing points/inputs [16]. From here onward, we will refer to them as knots. We will primarily examine only two sparse models, called the deterministic training conditional (DTC) and the fully independent conditional (FIC) approximations, using naming conventions established by [16]. However, it will be useful to discuss an additional two models (deterministic inducing conditional (DIC) and fully independent training conditional (FITC)) to better understand this class of knot‐based models [16]. We will explain the intuition behind these names in each of the relevant subsections.

Consider *K* knots denoted by $x^{†}={\left\{x_{k}^{†}\right\}}_{k=1}^{K}$. These are special input locations because they will induce the marginal covariances of all marginal function values. Reference [16] showed that many of the approximate GP posteriors commonly used in practice [7, 19, 20, 21] can be understood as resulting from different kinds of approximate priors on $\left(f_{\tilde{x}},f_{x},f_{x^{†}}\right)$. All approximate priors, $p\left(f_{\tilde{x}},f_{x},f_{x^{†}}\right)$, are defined so that

$$p_{\mathrm{GP}}\left(f_{\tilde{x}},f_{x},f_{x^{†}}\right)\approx p\left(f_{\tilde{x}},f_{x},f_{x^{†}}\right)=p\left(f_{\tilde{x}}|f_{x^{†}}\right)p\left(f_{x}|f_{x^{†}}\right)p_{\mathrm{GP}}\left(f_{x^{†}}\right),$$

where we use the subscript *GP* to specify the distribution implied by the full GP. All approximations require that $p\left(f_{x}|f_{x^{†}}\right)=\Pi_{i=1}^{N}p\left(f\left(x_{i}\right)|f_{x^{†}}\right)$, where $f_{x}=\left(f\left(x_{1}\right),\ldots ,f\left(x_{N}\right)\right)$. This results in a sparse precision matrix for $p\left(f_{x}|f_{x^{†}}\right)$, as well as for $p\left(f_{x}\right)$.

The four approximations we discuss result from two possible decisions for distributions, $p\left(f_{x}|f_{x^{†}}\right)$ and $p\left(f_{\tilde{x}}|f_{x^{†}}\right)$. These approximations were all discussed in [16]. We will reproduce essentially the same exposition for clarity. These four models result from either correcting the covariance matrix of $f_{x}|f_{x^{†}}$ to be the same as a full GP on the diagonal or by using the full GP conditional distribution for $f_{\tilde{x}}|f_{x^{†}}$. Table 1 shows the differences between the four sparse models we will consider in terms of whether or not the prior training and testing (co)variances match those of the full GP.

**Table 1:** Table showing whether or not certain marginal prior (co)variances implied by four sparse GP models match with the marginal prior (co)variances of the full GP

	Training covariances	Training variances	Test variances	Test covariances
DIC	NO	NO	NO	NO
DTC	NO	NO	YES	YES
FIC	NO	YES	YES	NO
FITC	NO	YES	YES	YES

### 3.1 Deterministic inducing conditional

The first and simplest approximation has been called the subset of regressors [18], predictive process model [1], and the DIC approximation [16]. We will use the terminology of [16]. The DIC model assumes that the latent function is *deterministic* once given the function values at the knots. Any *marginal* variance or covariance in the latent function is therefore *induced* by the knots. Let $\sum_{{\mathrm{xx}}^{\prime }}$ be the covariance matrix where the *ij*th element is given by $k_{\theta }\left(x_{i},x_{j}^{\prime }\right)$ and define $\Psi_{{\mathrm{xx}}^{\prime }}\equiv \sum_{{\mathrm{xx}}^{†}}\sum_{x^{†}x^{†}}^{-1}\sum_{x^{†}x^{\prime }}$. Then, the DIC approximation defines $p_{\mathrm{DIC}}\left(f_{x}|f_{x^{†}}\right)$ and $p_{\mathrm{DIC}}\left(f_{\tilde{x}}|f_{x^{†}}\right)$ as follows,

$$\begin{array}{l} f_{x}|f_{x^{†}}∼N\left(m_{x}+\sum_{xx^{†}}\sum_{x^{†}x^{†}}^{-1}\left(f_{x^{†}}-m_{x^{†}}\right),0\right)\\ f_{\tilde{x}}|f_{x^{†}}∼N\left(m_{\tilde{x}}+\sum_{\tilde{x}x^{†}}\sum_{x^{†}x^{†}}^{-1}\left(f_{x^{†}}-m_{x^{†}}\right),0\right). \end{array}$$

This, along with the marginal distribution $p\left(f_{x^{†}}\right)=N\left(m_{x^{†}},\sum_{x^{†}x^{†}}\right)$, which will be consistent across all models, implies the following marginal distributions for $f_{x}$ and $f_{\tilde{x}}$

$$\begin{array}{l} p_{\mathrm{DIC}}\left(f_{x}\right)=N\left(m_{x},\Psi_{\mathrm{xx}}\right)\\ p_{\mathrm{DIC}}\left(f_{\tilde{x}}\right)=N\left(m_{\tilde{x}},\Psi_{\tilde{x}\tilde{x}}\right). \end{array}$$

Reference [1] showed that this approximation is an optimal approximation to the full GP in the sense that, for any location, $\tilde{x}$, $E_{\mathrm{GP}}\left[\left.{\left(f\left(\tilde{x}\right)-g\left(f_{x^{†}}\right)\right)}^{2}\right|f_{x^{†}}\right]$ is minimized when

$$g\left(f_{x^{†}}\right)=m_{\tilde{x}}+\sum_{\tilde{x}x^{†}}\sum_{x^{†}x^{†}}^{-1}\left(f_{x^{†}}-m_{x^{†}}\right).$$

The expectation here is considered with respect to the full GP. Despite this optimal property, using this approximation tends to result in the underestimation of posterior function variances. This is because the prior GP variances for the DIC model are smaller than for the full GP. To observe this, note that, for the full GP, $V_{\mathrm{GP}}\left[f_{x}|f_{x^{†}}\right]=\sum_{\mathrm{xx}}-\Psi_{\mathrm{xx}}$. However, note that $V_{\mathrm{DIC}}\left[f_{x}|f_{x^{†}}\right]=\Psi_{\mathrm{xx}}$. Conditional variances are nonnegative, implying that the diagonal elements of $\Psi_{\mathrm{xx}}$ are smaller than the corresponding elements of $\sum_{\mathrm{xx}}$ [1]. The same is true of predictive variances at unobserved locations $\tilde{x}$.

### 3.2 Deterministic training conditional

The variance underestimation problem has led to two modifications to the DIC model. The first was discussed in ref. [19], which involved a different distribution for $p\left(f_{\tilde{x}}|f_{x^{†}}\right)$ resulting in a model they call projected latent variables. Reference [16] refers to this model as the DTC approximation. While the DIC model assumed all function values were deterministic given the function values at the knots, the DTC model assumes that this is only true of function values at *training* data input locations *x*. However, the function values at $\tilde{x}$ are not assumed to be deterministic conditional on the function values at the knots. Specifically, this approximation assumes that

$$f_{\tilde{x}}|f_{x^{†}}∼N\left(m_{\tilde{x}}+\sum_{\tilde{x}x^{†}}\sum_{x^{†}x^{†}}^{-1}\left(f_{x^{†}}-m_{x^{†}}\right),\sum_{\tilde{x}\tilde{x}}-\Psi_{\tilde{x}\tilde{x}}\right).$$

This is the exact distribution for $f_{\tilde{x}}|f_{x^{†}}$ if one were to use the full GP. Thus, $p_{\mathrm{DIC}}\left(f_{x}|f_{x^{†}}\right)=p_{\mathrm{DTC}}\left(f_{x}|f_{x^{†}}\right)$, but $p_{\mathrm{DIC}}\left(f_{\tilde{x}}|f_{x^{†}}\right)\neq p_{\mathrm{DTC}}\left(f_{\tilde{x}}|f_{x^{†}}\right)=p_{\mathrm{GP}}\left(f_{\tilde{x}}|f_{x^{†}}\right)$.

### 3.3 Fully independent conditional

The second modification to the DIC model was suggested independently in both [7, 21] and was called a sparse pseudoinput GP and a modified/bias‐corrected predictive process model in the two sources, respectively. Reference [16] refers to this model as the FIC approximation. In contrast to the DIC approximation, the FIC model does not assume that function values are deterministic conditional on the function values at the knots, but it does assume that function values are *conditionally independent* and have conditional variances matching that of the full GP.

This approximation makes modifications to both $p_{\mathrm{DIC}}\left(f_{x}|f_{x^{†}}\right)$ and $p_{\mathrm{DIC}}\left(f_{\tilde{x}}|f_{x^{†}}\right)$ compared to the distributions considered by the DIC model. FIC assumes the following conditional distributions for $f_{x}$ and $f_{\tilde{x}}$,

$$\begin{array}{l} f_{x}|f_{x^{†}}∼N\left(m_{x}+\sum_{{\mathrm{xx}}^{†}}\sum_{x^{†}x^{†}}^{-1}\left(f_{x^{†}}-m_{x^{†}}\right),\;\mathrm{diag}\left(\sum_{\mathrm{xx}}-\Psi_{\mathrm{xx}}\right)\right)\\ f_{\tilde{x}}|f_{x^{†}}∼N\left(m_{\tilde{x}}+\sum_{\tilde{x}x^{†}}\sum_{x^{†}x^{†}}^{-1}\left(f_{x^{†}}-m_{x^{†}}\right),\;\mathrm{diag}\left(\sum_{\tilde{x}\tilde{x}}-\Psi_{\tilde{x}\tilde{x}}\right)\right). \end{array}$$

This implies the following marginal distributions for $f_{x}$ and $f_{\tilde{x}}$,

$$\begin{array}{l} p_{\mathrm{FIC}}\left(f_{x}\right)=N\left(m_{x},\mathrm{diag}\left(\sum_{\mathrm{xx}}-\Psi_{\mathrm{xx}}\right)+\Psi_{\mathrm{xx}}\right)\\ p_{\mathrm{FIC}}\left(f_{\tilde{x}}\right)=N\left(m_{\tilde{x}},\mathrm{diag}\left(\sum_{\tilde{x}\tilde{x}}-\Psi_{\tilde{x}\tilde{x}}\right)+\Psi_{\tilde{x}\tilde{x}}\right). \end{array}$$

Thus, the FIC model assumes the same prior variances as the full GP, but the prior covariances are now different.

### 3.4 Fully independent training conditional

The final approximation we mention was first explicitly discussed in [16] and named the FITC model. This approximation modifies the FIC model so that the predictive covariances match that of the full GP. That is, $f_{\tilde{x}}|f_{x^{†}}$ is assumed to have the following distribution

$$f_{\tilde{x}}|f_{x^{†}}∼N\left(m_{\tilde{x}}+\sum_{\tilde{x}x^{†}}\sum_{x^{†}x^{†}}^{-1}\left(f_{x^{†}}-m_{x^{†}}\right),\sum_{\tilde{x}\tilde{x}}-\Psi_{\tilde{x}\tilde{x}}\right).$$

Thus, we find that $p_{\mathrm{FIC}}\left(f_{x}|f_{x^{†}}\right)=p_{\text{FITC}}\left(f_{x}|f_{x^{†}}\right)$, but $p_{\mathrm{FIC}}\left(f_{\tilde{x}}|f_{x^{†}}\right)\neq p_{\text{FITC}}\left(f_{\tilde{x}}|f_{x^{†}}\right)=p_{\mathrm{GP}}\left(f_{\tilde{x}}|f_{x^{†}}\right)$.

In the rest of the article, we will focus on the DTC and the FIC approximations. This is because the posterior distribution for $f_{\tilde{x}}$ resulting from the DTC prior can be derived as the marginal of an optimal posterior approximation to $p_{\mathrm{GP}}\left(f_{\tilde{x}},f_{x},f_{x^{†}}|y\right)$ in a sense that we will discuss in Section 4.1. In addition, we are primarily interested in marginal predictive distributions, which are the same for the FIC and FITC models.

## 4 VARIATIONAL INFERENCE

In this section, we discuss variational inference (VI) in a general context, and in Section 4.1, we discuss the particular approximation relevant to GP regression. Variational inference is an analytical, optimization‐based method for approximating probability distributions [3]. The goal of VI is to approximate a potentially intractable distribution *P* defined on Z with a *variational distribution Q*. It is standard to assume that *P* and *Q* have probability densities of *p* and *q*, respectively, with respect to some probability measure *μ*. We then define our objective function to be

$$D\left(Q\|P\right)=\int_{Z}q\left(z\right)\log\frac{q\left(z\right)}{p\left(z\right)}d\mu \left(z\right),$$

the Kullback‐Leibler (KL) divergence of *P* with respect to *Q*. We will consider this objective function in the context of trying to approximate posterior distributions of some parameters *Z* given observed data, *Y*. Going forward, we will write *p*(*z*| *y*) instead of *p*(*z*) to make this explicit.

The KL divergence above is often not analytically tractable. Ref. [13], however, showed that minimizing the above KL divergence is equivalent to maximizing a lower bound on the log‐likelihood, commonly called the *evidence lower bound* (or ELBO). We reproduce this derivation as it is shown in [3]. The KL divergence can be written as

$$\begin{array}{cc} D\left(Q\|P\right) & =E\left[\log q\left(z\right)\right]-E\left[\log p\left(z,y\right)\right]+E\left[\log p\left(y\right)\right]\\ & =E\left[\log q\left(z\right)\right]-E\left[\log p\left(z,y\right)\right]+\log p\left(y\right), \end{array}$$

where expectations are with respect to the distribution *Q*. By rearranging terms, we see that

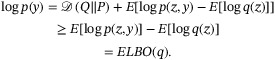



Thus, we see that, by maximizing *ELBO*(*q*) with respect to the distribution *q*, we minimize $D\left(Q\|P\right)$ as log *p*(*y*) is not a function of *q*. For example, when log *p*(*y*) = *E* [log *p*(*x*, *y*)] − *E* [log *q*(*x*)], it must be that $D\left(Q\|P\right)=0$, which implies that *P* = *Q*. In general, any arbitrary *Q* need not result in an analytically tractable expression for the ELBO. However, typically, *q*(*z*) and *p*(*z*, *y*) will have analytical expressions, but the expectations may be challenging or impossible to compute analytically.

### 4.1 Variational inference in sparse GPs

Ref. [22] showed how the approximate posterior, $p_{\mathrm{DTC}}\left(f_{\tilde{x}}|y\right)$, can be derived by using a predictive distribution that can be written as $\int p_{\mathrm{GP}}\left(f_{\tilde{x}}|f_{x^{†}}\right)h^{*}\left(f_{x^{†}}\right){\mathrm{df}}_{x^{†}}$, where $h^{*}\left(f_{x^{†}}\right)=p_{\mathrm{DTC}}\left(f_{x^{†}}|y\right)$ is the marginal distribution resulting from the optimal variational approximation to $p_{\mathrm{GP}}\left(f_{x},f_{x^{†}}|y\right)$ in the class of distributions, Q, with densities *q* that can be written as

$$q\left(f_{x},f_{x^{†}}\right)=p_{\mathrm{GP}}\left(f_{x}|f_{x^{†}}\right)h\left(f_{x^{†}}\right).$$

Here, note that *h* is considered to be a “free form” variational distribution for $f_{x^{†}}$, meaning that it is not restricted to be from any specific distributional family. Reference [19] derives essentially the same result while pursuing the goal of finding and justifying a sparse likelihood approximation. We reproduce essentially the same derivation of the optimal variational distribution and the corresponding ELBO in Appendix A. The ELBO arising from this optimal variational approximation is given by

$$\begin{array}{cc} & \text{ELBO}\left(q^{*}\right)=\log\left[N\left(y;\;m_{x},\Psi_{\mathrm{xx}}+\tau^{2}I\right)\right.\\ & \;\left.-\frac{1}{2\tau^{2}}\mathrm{Tr}\left(V_{\mathrm{GP}}\left[f_{x}|f_{x^{†}}\right]\right)\right], \end{array}$$

where we use q^*^ to denote the optimal variational d istribution.

Using the optimal variational approximation and ELBO, derivatives of the ELBO are taken with respect to covariance parameters and the knots. These derivatives can be used to optimize the ELBO with a gradient‐based optimization routine. In keeping with terminology in [2], we will refer to the model resulting from this variational approximation in combination with using the ELBO for model selection the *variational free energy* (VFE) model.

### 4.2 Knot selection using the ELBO

The ELBO is an appealing objective function for knot selection because it never decreases with an addition of a new knot [2, 22]. To gain some intuition for this, first recall that maximizing the ELBO is equivalent to minimizing the KL divergence between the approximate and the full posterior. At a high level, adding knots results in a prior covariance matrix in the sparse model that better approximates the prior covariance matrix in the full GP model, and so the, KL divergence between the two posteriors will be smaller. More concretely, note that the ELBO is the sum of two terms: the first is the marginal likelihood of the DTC/DIC model, and the second is a strictly negative term consisting of the negative (scaled) sum of the conditional variances of $f_{x}$ gives $f_{x^{†}}$ according to the full GP. The first term measures how well the model fits the data, but it does not depend at all on the full GP posterior that we are trying to approximate. The second term does not depend on the data, but it does depend on the full GP posterior (through the full GP prior). Thus, it is the second term that must encourage the approximate posterior to resemble that of the full GP. Indeed, $V_{\mathrm{GP}}\left[f_{x}|f_{x^{†}}\right]$ can only decrease or remain constant as the number of knots grows. The fact that the change in the second term in the ELBO offsets any decrease in the first term is nontrivial, and we refer curious readers to [2] for the proof.

**Figure 1:**
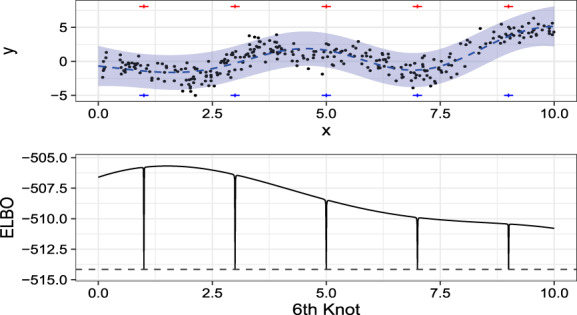
The top panel shows the fit from a five knot VFE model, while the bottom panel shows the ELBO values as a function of the location of a single, sixth knot with first five knots (blue and red +) held fixed. The ELBO value for the model without the sixth knot is plotted as a horizontal dashed line

Unfortunately, adding knots OAT can be tricky in practice. An intuitively reasonable method for selecting knots and covariance parameters might be to first initialize a small set of knots and covariance parameter values. One could then consider adding a knot followed by continuous optimization of the ELBO with respect to either of the covariance parameters exclusively or the covariance parameters and the added knot. However, Figure 1 shows a phenomenon discussed in [2] where spikes in the ELBO exist whenever a new knot is placed directly on top of a previously existing knot. Furthermore, [2] also notes that the addition of a small noise variance of *f*(*x*), often necessary for numerical stability of matrix inverses, results in a widening of these spikes. This causes suboptimal local maxima, which can be sufficient to disrupt an optimization algorithm.

Reference [22] suggested the possibility of greedily adding a knot by choosing the value that maximized improvement to the ELBO over some small random sample of observed data locations. While this may often work reasonably well in practice, there may be more efficient ways of searching for the observed data locations. Reference [8] proposed using Bayesian optimization to efficiently propose a new knot, which is then optimized alongside covariance parameters holding previous knots fixed using gradient‐based methods. Reference [8] showed, that compared to optimization of all knots simultaneously, their OAT knot selection algorithm was often at least as accurate but was usually many times faster. Thus, we propose using a slightly modified version of the OAT method to select knots using the ELBO from the VFE method as the objective function. Note that this requires a covariance function that is differentiable in the knot locations. The only difference between our implementation here and the implementation in ref. [8] is that we do not condition on the values of the ELBO when the new knot is located in the same spot as an existing knot in the Bayesian optimization knot proposal function. As in ref. [8], we refer to the OAT algorithm that uses Bayesian optimization for the proposal function as the OAT‐BO algorithm. Because we are primarily concerned with regression problems, in which the true latent function can reasonably be assumed to be fairly smooth, we consider using covariance functions, resulting in smooth GP realizations. Furthermore, our knot selection algorithm requires that the covariance function is at least once differentiable in the knot locations. Thus, in every application, we use the squared exponential covariance function, $k_{\theta }\left(x,x^{\prime }\right)=\sigma^{2}e^{\frac{-{\left\|x-x^{\prime }\right\|}^{2}}{2\ell^{2}}}$. However, one could certainly consider using any other covariance function that is once differentiable in the knot locations.

**Figure 2:**
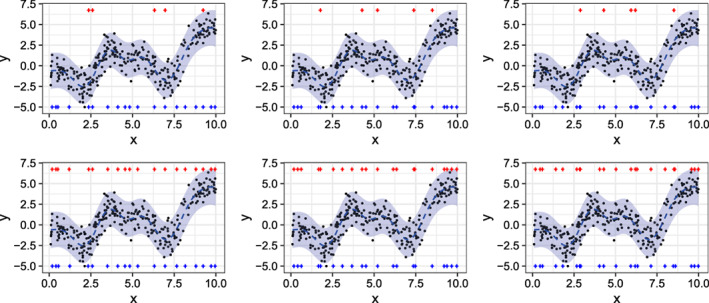
VFE model fits to a 300 observation‐synthetic, one‐dimensional regression using the OAT‐BO algorithm (top row) and refinements of the placed knots through simultaneous optimization (bottom row). Initial knots (red+) and final knots (blue+) are shown on the top and bottom of each plot, respectively

As an illustrative example, Figure 2 shows results on a synthetic, one‐dimensional regression problem with 300 observations. We see that the OAT‐BO algorithm selects knots roughly uniformly across the x‐axis and selects roughly the same numbers of knots. We also see that the refinements to the knots placed by the OAT‐BO algorithm in the bottom row are minimal. Thus, in this case, the OAT‐BO algorithm appears to have placed knots near a local maximum. The predictions and uncertainties from each fit looks nearly identical.

## 5 EXPERIMENTS

In this section, we compare the OAT‐BO algorithm to several alternatives for knot selection on three publicly available datasets. In all experiments, we test the OAT‐BO algorithm in a VFE model, the OAT‐BO algorithm in an FIC model where the model selection objective function is the marginal likelihood, the OAT algorithm using the best‐of‐random‐subset (abbreviated as “RS”) proposal as in [8] in a VFE model, and a refinement of the fit of the VFE model selected through the OAT‐BO algorithm by simultaneously optimizing all knots and covariance parameters. In every model, we add a small nugget to the latent function to ensure that the relevant inverses are numerically stable. Knots for all models, except for the VFE refinement, were initialized using k‐means clustering. Covariance parameters in all models were initialized to the same values. The maximum number of knots allowed by all OAT algorithms was set to 80. Furthermore, the number of knots in the simultaneously optimized models were set to be equal to the number found by the OAT‐BO algorithm. Finally, all gradient‐based optimizations were performed using ADADELTA [26], as in [8]. R [17] code to reproduce all results in this work is available as a package called sparseRGPs at https://github.com/nategarton13/sparseRGPs.

We use the same, slightly modified versions of canonical performance metrics in ref. [8], reflecting the fact that we are only interested in marginal predictive densities. The two main metrics we consider are common to all of our experiments. The first metric is the median negative log‐probability (MNLP), which is calculated as

$$\text{MNLP}={\text{median}}_{i\in 1,\ldots ,N_{\text{test}}}\left\{-\log p\left({\tilde{y}}_{i}|x^{†},\hat{\theta },y\right)\right\}.$$

Lower MNLP values correspond to more accurate marginal predictive densities. The second metric we calculate is standardized root mean squared error (SRMSE), which is calculated by averaging the squared differences between predictions and the test data and is normalized by the sample standard deviation on the test set. That is,

$$\text{SRMSE}=\sigma_{\tilde{y}}^{-1}\sqrt{\frac{1}{N_{\text{test}}}\sum_{i=1}^{N_{\text{test}}}{\left(E\left[f\left({\tilde{x}}_{i}\right)|Y\right]-{\tilde{y}}_{i}\right)}^{2}},$$

where $\sigma_{\tilde{y}}^{2}=\frac{1}{N_{\text{test}}-1}\sum_{i=1}^{N_{\text{test}}}{\left({\tilde{y}}_{i}-\underline{\tilde{y}}\right)}^{2},\;\underline{\tilde{y}}=\frac{1}{N_{\text{test}}}\sum_{i=1}^{N_{\text{test}}}{\tilde{y}}_{i}$, and $\tilde{y}$ is the vector of test set target values. In addition, we provide the time in seconds required to train each model and the final number of knots used for each.

### 5.1 Boston housing data

The first dataset that we consider is the Boston housing dataset1
http://lib.stat.cmu.edu/datasets/boston
 [10]. As in [8], we use “% lower status of the population”, “average number of rooms per dwelling”, and “pupil‐teacher ratio by town” to predict the median value of owner‐occupied homes. We also removed observations where the median value was less than $50 000, leaving 490 observations. For each of five runs, we randomly selected ≈80% of the data for training and used the remaining 20% for prediction. In addition to the four models mentioned in Section 5, this dataset is small enough that we can easily fit the full GP. In addition, to more accurately provide results for what is currently common practice, we also provide results for a VFE model where knots and covariance parameters are found by simultaneous optimization, and knots are initialized with k‐means clustering. Table 2 provides a summary of the models that we fit for this dataset.

**Table 2:** List of models fit to the Boston housing data

Model	Knot selection	Approximation	Knot init.
FGP	—	—	—
OBVk	OAT‐BO	VFE	k‐means
ORVk	OAT‐RS	VFE	k‐means
OBFk	OAT‐BO	FIC	k‐means
SVk	Simult.	VFE	k‐means
SVO	Simult.	VFE	OAT‐BO

*Note*: The first model in the table is a full GP.

In addition to MNLP and SRMSE, we also measure the difference between predictions resulting from the full GP and those resulting from the sparse models. For this, we use the average univariate KL divergence (AUKL) (or its log value) between the predictive density from the full GP and that of each sparse model. We calculate this as

$$\begin{array}{cc} \text{AUKL} & =\frac{1}{N_{\text{test}}}\sum_{i=1}^{N_{\text{test}}}\int p_{\text{full}}\left(f\left({\tilde{x}}_{i}\right)|\hat{\theta },y\right)\\ & \;\times \log\frac{p_{\text{full}}\left(f\left({\tilde{x}}_{i}\right)|\hat{\theta },y\right)}{p_{\text{sparse}}\left(f\left({\tilde{x}}_{i}\right)|x^{†},\hat{\theta },y\right)}\mathrm{df}\left({\tilde{x}}_{i}\right). \end{array}$$

**Figure 3:**
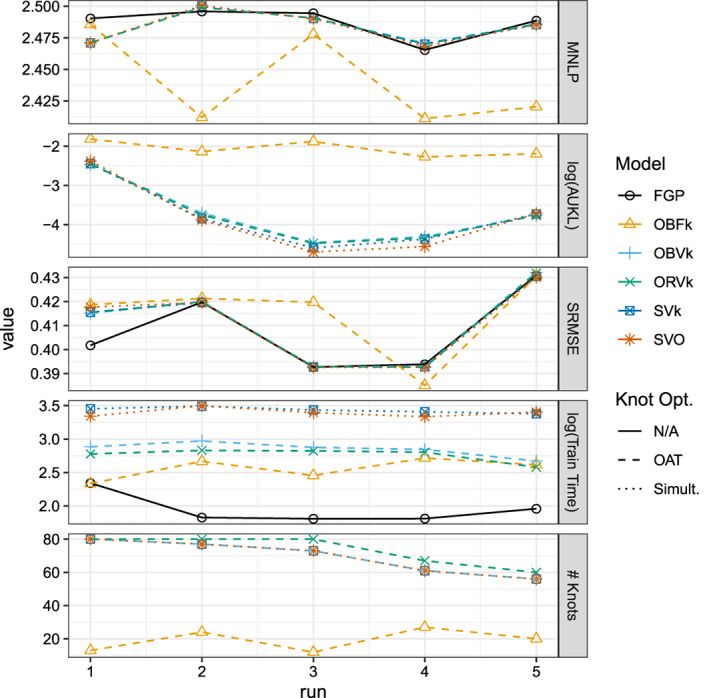
Results on the Boston housing dataset for five randomly sampled training and test sets. Model enumeration corresponds to Table 2

Figure 3 shows results from each model on each random test set of the Boston data. Broadly speaking, we see close agreement across all five runs of the accuracy measures for the VFE and the full GP models. However, we see that the simultaneously optimized VFE models tend to take two or three times longer to fit. Any differences between using the BO and the RS proposal seem to be minimal. The FIC model had the largest differences between the other models. For one, it tends to choose models with fewer than half as many knots as the VFE models. As one might expect, this corresponds to substantially different predictive distributions compared to the full GP as measured by the (log base 10) AUKL. However, it is unclear if the FIC model makes less‐accurate point predictions as, other than on the third run, the SRMSE values are competitive with each of the other models. Furthermore, the FIC MNLP values are smallest for all but the first run, where MNLP is similar to the other models.

### 5.2 Airfoil data

In the second experiment, we use the Airfoil self‐noise dataset,2
https://archive.ics.uci.edu/ml/datasets/Airfoil+Self-Noise
 which is available from the University of California, Irvine (UCI) machine learning repository [6]. The goal is to predict a component of the overall noise, measured in decibels, generated by the airfoil blade of a certain aircraft from five continuous predictors [9]. We fit the same set of models as in the Boston experiment, which are listed in Table 2.

**Figure 4:**
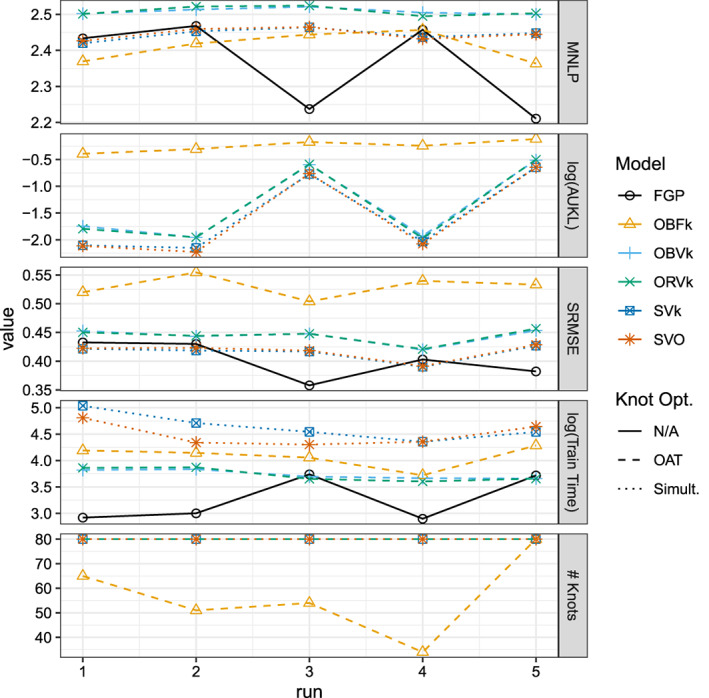
Results on the Airfoil dataset for five randomly sampled training and test sets. Model enumeration corresponds to Table 2

Figure 4 shows results from each model on each random test set of the Airfoil data. Here, results differ slightly from those on the Boston housing data. We see consistent results for the VFE models chosen via OAT‐BO and OAT‐RS methods, but simultaneous optimization seems to result in relatively small, but consistent, improvements over the OAT methods. This improvement comes at an additional computational cost, which is occasionally reduced by initializing knots to those in the VFE model chosen by the OAT‐BO algorithm. The average time to fit the VFE model with the OAT‐BO algorithm was close to 10% of the average time required by the simultaneously optimized VFE model initialized with k‐means. Interestingly, while we see that the FIC model is again competitive with respect to the MNLP metric, it now performs consistently worse in terms of SRMSE, explaining roughly 0.5^2^ − 0.45^2^ = 5% to 0.55^2^ − 0.45^2^ = 10% less variability in the target variable than the VFE models selected using the OAT algorithm.

### 5.3 Combined cycle power plant data

For our third and final experiment, we consider the Combined Cycle Power Plant (CCPP) dataset,3
https://archive.ics.uci.edu/ml/datasets/Combined+Cycle+Power+Plant
 which is available from the UCI machine learning repository [6]. The goal is to predict the full load power output of a combined cycle power plant [14, 24]. The dataset consists of 9568 observations of the target variable, power output, along with four other predictor variables. We randomly split the data five times ≈50/50 into training and testing sets and provide results for a subset of the models considered in the previous experiments. We do not fit the full GP, nor do we fit VFE models with simultaneous knot optimization where the knot initialization was performed with k‐means due to time constraints. As such, we do not compute the AUKL measure here. Table 3 summarizes the four different model fits on each experimental run. Model enumeration is kept consistent with the previous experiments for clarity.

**Table 3:** List of models fit to the CCPP dataset

Model	Knot selection	Approximation	Knot Init.
OBVk	OAT‐BO	VFE	k‐means
ORVk	OAT‐RS	VFE	k‐means
OBFk	OAT‐BO	FIC	k‐means
SVO	Simult.	VFE	OAT‐BO

**Figure 5:**
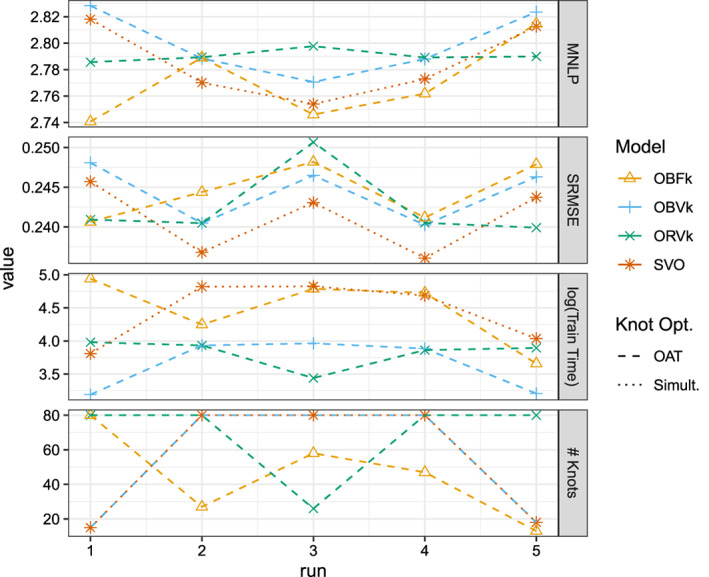
Results on the CCPP dataset for five randomly sampled training and test sets. Model enumeration corresponds to Table 3

Figure 5 shows the results of the four models for the five experimental runs. Overall, the four models are similarly accurate, with different models achieving MNLP values between roughly 2.74 and 2.83 and SRMSE values between roughly 0.23 and 0.25 across all five runs. Consistent with results on the Airfoil data, we see that simultaneous optimization of the knots found by the OAT‐BO algorithm in the VFE model results in consistent improvements in the MNLP and SRMSE values. When the OAT‐BO algorithm selects the full 80 possible knots, training time is approximately six to seven times slower when performing the simultaneous optimization in the VFE model. Surprisingly, despite the FIC model often having a smaller number knots than the VFE models, training times tended to be roughly comparable to the simultaneous optimization in the VFE model.

## 6 DISCUSSION

We have tested the OAT knot selection algorithm proposed in ref. [8] to choose the number and locations of knots in the approximate GP regression model proposed by ref. [22]. We compared the results on three benchmark regression tasks and found that using the OAT algorithm is always several times faster and results in predictions that are competitive with the simultaneous optimization of knots.

Reference [8] discussed why the OAT algorithm is typically faster than simultaneous optimization when the objective function is the marginal likelihood, but the same rationale applies here, namely, that gradient evaluations cost $O\left({\mathrm{dNK}}^{3}\right)$ floating point operations for simultaneous optimization and only $O\left({\mathrm{dNK}}^{2}\right)$ for the OAT algorithm. This difference is even more noticeable as *d* grows, especially for datasets with large *N*. The OAT algorithm does incur additional costs due to the knot proposal function, and OAT usually requires a greater absolute number of gradient ascent steps. However, these costs are usually relatively small in practice.

Furthermore, [8] commented that the simultaneous optimization of knots with the marginal likelihood as the objective function could result in undesirable solutions where several knots serve practically no function. This behavior was also discussed in ref. [2]. OAT has consistently been able to circumvent this problem, and this offers a partial explanation as to why OAT may provide competitive or better accuracy when using the marginal likelihood as the objective. However, it is notable that this issue seems far less prevalent when the ELBO is used as the objective function. Therefore, why OAT seems to be competitive with simultaneous optimization of knots when variational inference is used is less clear. With that being said, we make a couple of remarks. First, OAT can be viewed as a kind of forward selection algorithm of basis functions in a Bayesian linear nonparametric regression, and so, the extent to which forward selection algorithms are successful for finding predictive linear regression models is likely to be similar here. Second, there are likely many good configurations of knots resulting in very similar predictive distributions. We observe this in Figure 2, where none of the knot configurations were the same between each model, but model fits were nearly indistinguishable. Thus, it seems that significant sophistication may be unnecessary in knot selection algorithms.

We did see that it is sometimes possible to slightly improve the models found using the OAT algorithm by refining the knot locations through simultaneous optimization. Thus, time permitting, one could consider using the OAT algorithm as a way to obtain a good initialization. Furthermore, while we initialized covariance parameters identically in all models for the sake of comparability, we suspect that it would be much faster to initialize covariance parameters in those found by OAT in case OAT is used as an initialization step.

Interestingly, we did not see substantial differences between using the RS proposal mechanism and the BO proposal mechanism. This is consistent with what was found in ref. [8] when the marginal likelihood was used as the objective function. We do find some evidence that, when a model with few knots can perform well in, for example, the Boston housing, using the BO proposal tended to select slightly sparser models than the RS proposal. This may also have been true of the CCPP data as, there, the average number of knots selected by the OAT‐BO proposal was smaller than the average number of knots selected by the OAT‐RS proposal, but this was not consistent across runs. The VFE models using the BO proposal had, on average, four fewer final knots than using the RS proposal. This makes sense as the Bayesian optimization should more efficiently search candidate knots and avoid local maxima. However, in the Airfoil data, where 80 knots were always selected in the OAT models, accuracy was indistinguishable between the RS and the BO proposals. Reference [8] suggested some reasons why this BO proposal may not outperform the RS proposal, such as the possibility that the Bayesian optimization spends too much time exploring local maxima or that finding a global maximum for a new knot tends to result in a final set of knots that is too sparse or clearly suboptimal.

Finally, we also showed how the VFE models compared to the FIC models where optimization was performed through the OAT‐BO algorithm. When the objective function is the log‐marginal likelihood, the OAT algorithm tends to reliably avoid placing knots directly on top of each other as has been discussed by, for example, ref. [2]. The OAT‐BO algorithm often chooses sparser FIC models than VFE. Interestingly, this did not consistently result in either faster training time or reduced accuracy by the measures we considered. We do, however, see that the FIC model does not approximate the full GP posterior nearly as well as the VFE model does, as measured by the KL divergence between the predictive distributions coming from the full GP and the sparse models. The fact that this occurs, but that MNLP and SRMSE values can be competitive with the full GP and the VFE models, suggests that the FIC approximation has utility beyond its ability to mimic a full GP.

With that being said, if the goal of the modeler is to efficiently estimate predictive densities resembling a full GP, then, like ref. [2], our recommendation is to use the VFE approximation over the FIC model. The reason for this is that training time in the VFE models is usually at least as short as it is for FIC models, but the VFE models appear to more reliably obtain (S)RMSE and MNLP values competitive with a full GP. Furthermore, even when FIC models result in good accuracy on the test set, the predictive densities tend to differ from the full GP more than the VFE models.

## CONFLICT OF INTEREST

The authors declare no conflict of interests.

## Appendix A OPTIMAL VARIATIONAL DISTRIBUTION DERIVATION

Here, we reproduce essentially the same derivation of the optimal variational distribution and the corresponding ELBO from ref. [23]. Note that by “optimal variational distribution,” we mean that, for the class of approximate posteriors that we consider and for a fixed set of knots, we can find the exact approximate posterior that maximizes the ELBO. Our minor modification to the derivation in ref. [23] allows one to arrive at the same approximation in a slightly simpler way. We may simply modify our target posterior distribution to be $p_{\mathrm{GP}}\left(f_{\tilde{x}},f_{x},f_{x^{†}}|y\right)$ and use a modified class of distributions, ℛ, with densities *r* that can be written as

$$r\left(f_{\tilde{x}},f_{x},f_{x^{†}}\right)=p_{\mathrm{GP}}\left(f_{\tilde{x}},f_{x}|f_{x^{†}}\right)h\left(f_{x^{†}}\right).$$

We can then write the ELBO as follows

This is the same ELBO as derived by [22], and so, the same arguments apply to derive the optimal distribution *h*^*^. The remaining work is replicated from [23] with some minor notational differences. First, we evaluate $\int p_{\mathrm{GP}}\left(f_{x}|f_{x^{†}}\right)\log p\left(y|f_{x}\right){\mathrm{df}}_{x}$ analytically as follows,

$$\begin{array}{ll} & \int p_{\mathrm{GP}}\left(f_{x}|f_{x^{†}}\right)\log p\left(y|f_{x}\right){\mathrm{df}}_{x}\\ & \;=E_{p}\left[\left.-\frac{N}{2}\log\left(2{\pi \tau }^{2}\right)-\frac{1}{2\tau^{2}}\sum_{i=1}^{N}{\left(y_{i}-f\left(x_{i}\right)\right)}^{2}\right|f_{x^{†}}\right]\\ & \;=-\frac{N}{2}\log\left(2{\pi \tau }^{2}\right)-\frac{1}{2\tau^{2}}E_{p}\\ & \;\left[\left.\sum_{i=1}^{N}{\left(\left[y_{i}-\underline{m}\left(x_{i}\right)\right]-\left[f\left(x_{i}\right)-\underline{m}\left(x_{i}\right)\right]\right)}^{2}\right|f_{x^{†}}\right]\\ & \;=-\frac{N}{2}\log\left(2{\pi \tau }^{2}\right)-\frac{1}{2\tau^{2}}\\ & \;\left[\sum_{i=1}^{N}{\left(y_{i}-\underline{m}\left(x_{i}\right)\right)}^{2}+\mathrm{Tr}\left(\sum_{\mathrm{xx}}-\sum_{{\mathrm{xx}}^{†}}\sum_{x^{†}x^{†}}^{-1}\sum_{x^{†}x}\right)\right]\\ & \;\equiv \log G\left(f_{x^{†}},y\right), \end{array}$$

where $\underline{m}\left(x_{i}\right)\equiv E_{p}\left[f\left(x_{i}\right)|f_{x^{†}}\right]$, and expectations are with respect to $p_{\mathrm{GP}}\left(f_{x}|f_{x^{†}}\right)$. In the future, it will be useful to note that $\log G\left(f_{x^{†}},y\right)=\log N\left(y;\underline{m}\left(x\right),\tau^{2}I\right)-\frac{1}{2\tau^{2}}\mathrm{Tr}\left(V\left[f_{x}|f_{x^{†}}\right]\right)$.

We then note that

$$\text{ELBO}\left(r\right)=\int h\left(f_{x^{†}}\right)\log\frac{G\left(f_{x^{†}},y\right)p_{\mathrm{GP}}\left(f_{x^{†}}\right)}{h\left(f_{x^{†}}\right)}{\mathrm{df}}_{x^{†}}.$$

We now look for a distribution *h* that achieves an upper bound on the ELBO. We can do this, as explained by ref. [23], by using Jensen's inequality to see that

where recall that we have defined $\Psi_{\mathrm{xx}}=\sum_{x^{†}x}\sum_{x^{†}x^{†}}^{-1}\sum_{{\mathrm{xx}}^{†}}$. Jensen's inequality becomes an equality when $\frac{G\left(f_{x^{†}},y\right)p_{\mathrm{GP}}\left(f_{x^{†}}\right)}{h\left(f_{x^{†}}\right)}$ is a constant, and this occurs when $h\left(f_{x^{†}}\right)\propto N\left(y;\underline{m}\left(x\right),\tau^{2}I\right)p\left(f_{x^{†}}\right)$. The term on the right‐hand side of the proportionality sign can be viewed as a joint distribution for $\left(Y,f_{x^{†}}\right)$ resulting from a Gaussian likelihood with a Gaussian prior on the mean. Furthermore, note that the analytically tractable posterior for $f_{x^{†}}$, given *y* in this model, is proportional to the joint distribution and thus works as a choice for $h\left(f_{x^{†}}\right)$. Thus, we set

$$\begin{array}{cc} & h^{*}\left(f_{x^{†}}\right)=N\left(m_{x^{†}}+\sum_{x^{†}x}{\left[\Psi_{\mathrm{xx}}+\tau^{2}I\right]}^{-1}\left(y-m_{x}\right)\right.\\ & \;\left.\sum_{x^{†}x^{†}}-\sum_{x^{†}x}{\left[\Psi_{\mathrm{xx}}+\tau^{2}I\right]}^{-1}\sum_{{\mathrm{xx}}^{†}}\right). \end{array}$$

Using the fact that this choice for *h* is, in fact, the posterior distribution for the model

$$\begin{array}{l} Y|f_{x^{†}}∼N\left({\underline{m}}_{x},\tau^{2}I\right)\\ f_{x^{†}}∼N\left(m_{x^{†}},\sum_{x^{†}x^{†}}\right), \end{array}$$

with marginal likelihood $Y∼N\left(m_{x},\Psi_{\mathrm{xx}}+\tau^{2}I\right)$, it is trivial to show that this choice of *h* achieves the upper bound on the ELBO and is therefore optimal. Moreover, we have shown that the ELBO is, in fact, equal to

$$\begin{array}{cc} & \text{ELBO}\left(r^{*}\right)=\log\left[N\left(y\;;\;m_{x},\Psi_{\mathrm{xx}}+\tau^{2}I\right)\right.\\ & \left.\;-\frac{1}{2\tau^{2}}\mathrm{Tr}\left(V\left[f_{x}|f_{x^{†}}\right]\right)\right], \end{array}$$

where we use *r*^*^ to denote the optimal variational distribution.

## References

[1] S. Banerjee et al., Gaussian predictive process models for large spatial data sets, J. R. Stat. Soc.: Ser. B (Stat. Methodol.) 70(4) (2008), 825–848. 10.1111/j.1467-9868.2008.00663.x PMC2741335 19750209

[2] M. Bauer , M. van der Wilk , and C. E. Rasmussen , Understanding probabilistic sparse Gaussian process approximations, in Advances in Neural Information Processing Systems 29, Lee D. D. et al., Eds., Curran Associates, Inc., Red Hook, NY, 2016, 1533–1541. http://papers.nips.cc/paper/6477‐understanding‐probabilistic‐sparse‐gaussian‐process‐approximations.pdf.

[3] D. M. Blei , A. Kucukelbir , and J. D. McAuliffe , Variational inference: A review for statisticians, J. Amer. Statist. Assoc. 112(518) (2017), 859–877. 10.1080/01621459.2017.1285773.

[4] Y. Cao et al., Efficient optimization for sparse Gaussian process regression, Adv. Neural Inform. Process. Syst. 26(2013), 1097–1105.

[5] A. Datta et al., Hierarchical nearest‐neighbor Gaussian process models for large geostatistical datasets, J. Amer. Statist. Assoc. 111(514) (2016), 800–812. 10.1080/01621459.2015.1044091 PMC5927603 29720777

[6] D. Dua and C. Graff , *UCI machine learning repository*, 2017, available at http://archive.ics.uci.edu/ml

[7] A. O. Finley et al., Improving the performance of predictive process modeling for large datasets, Comput. Statist. Data Anal. 53(8) (2009), 2873–2884. 10.1016/j.csda.2008.09.008. PMC2743161 20016667

[8] N. Garton , J. Niemi , and A. Carriquiry , Knot selection in sparse Gaussian processes, *arXiv preprint arXiv:2002.09538*, 2020. 10.1002/sam.11459 PMC7386924 32742538

[9] R. L. González , Neural networks for variational problems in engineering, Ph.D. Thesis, Tech. Univ. of Catalonia, 2008.

[10] D. Harrison and D. Rubinfeld , Hedonic prices and the demand for clean air, Econ. Manage. 5 (1978), 81–102.

[11] J. Hensman , A. Matthews , and Z. Ghahramani , *Scalable variational Gaussian process classification*, Proceedings of the Eighteenth International Conference on Artificial Intelligence and Statistics (Guy Lebanon and S. V. N. Vishwanathan, eds.), San Diego, California, Volume 38 of Proceedings of Machine Learning Research, 2015, pp. 351‐360. available at http://proceedings.mlr.press/v38/hensman15.html.

[12] Hernandez‐Lobato, D. and J. M. Hernandez‐Lobato , *Scalable Gaussian process classification via expectation propagation*, Proceedings of the 19th International Conference on Artificial Intelligence and Statistics (A. Gretton and C. C. Robert, eds.), PMLR, Cadiz, Spain, volume 51 of Proceedings of Machine Learning Research, 2016, pp. 168–176. available at http://proceedings.mlr.press/v51/hernandez-lobato16.html

[13] M. I. Jordan et al., An introduction to variational methods for graphical models, Mach. Learn. 37(2) (1999), 183–233.

[14] H. Kaya , P. Tüfekci , and F. S. Gürgen , *Local and global learning methods for predicting power of a combined gas & steam turbine*, Proceedings of the International Conference on Emerging Trends in Computer and Electronics Engineering (ICETCEE), 2012, pp. 13–18.

[15] A. Naish‐Guzman and S. Holden , The generalized FITC approximation, Adv. Neural Inform. Process. Syst. 28 (2008), 1057–1064.

[16] J. Quiñonero‐Candela and C. E. Rasmussen , A unifying view of sparse approximate Gaussian process regression, J. Mach. Learn. Res. 6 (2005), 1939.

[17] R Core Team , R: A language and environment for statistical computing, R Foundation for Statistical Computing, Vienna, Austria, 2017 https://www.R-project.org/.

[18] C. E. Rasmussen and C. K. Williams , Gaussian processes for machine learning. Cambridge, MA: MIT Press, 2006.

[19] M. Seeger , C. Williams , and N. Lawrence , Fast forward selection to speed up sparse Gaussian process regression, Artif. Intell. Stat. 9 (2003).

[20] A. J. Smola and P. L. Bartlett , Sparse greedy Gaussian process regression, Adv. Neural Inform. Process. Syst. 14 (2001), 619–625.

[21] Snelson, E. and Z. Ghahramani , 2006: Sparse Gaussian processes using pseudo‐inputs. Advances in neural information processing systems 18 *,* Weiss Y., Schölkopf B., and Platt J. C., Eds., MIT Press, 1257–1264, available at http://papers.nips.cc/paper/2857-sparse-gaussian-processes-using-pseudo-inputs.pdf

[22] M. Titsias , Variational learning of inducing variables in sparse Gaussian processes, Artif. Intell. Stat. 5 (2009a), 567–574.

[23] M. Titsias , Variational model selection for sparse Gaussian process regression, University of Manchester, 2009b.

[24] P. Tüfekci , Prediction of full load electrical power output of a base load operated combined cycle power plant using machine learning methods, Int. J. Electr. Power Energy Syst. 60(2014), 126–140.

[25] C. K. Williams and M. Seeger , *Using the Nyström method to speed up kernel machines,* Advances in Neural Information Processing Systems, 2001, pp. 682–688.

[26] M. D. Zeiler , *ADADELTA: an adaptive learning rate method*, 2012, available at https://arxiv.org/abs/1212.5701

